# A Functional γδTCR/CD3 Complex Distinct from γδT Cells Is Expressed by Human Eosinophils

**DOI:** 10.1371/journal.pone.0005926

**Published:** 2009-06-17

**Authors:** Fanny Legrand, Virginie Driss, Gaëtane Woerly, Sylvie Loiseau, Emmanuel Hermann, Jean-Jacques Fournié, Laurent Héliot, Virginie Mattot, Fabrice Soncin, Marie-Lise Gougeon, David Dombrowicz, Monique Capron

**Affiliations:** 1 Inserm U547, Lille, France; 2 Université Lille - Nord de France, Lille, France; 3 Institut Pasteur de Lille, Lille, France; 4 Inserm, U 563, Toulouse, France; 5 CNRS UMR8161, Institut de Biologie de Lille, Lille, France; 6 Institut Pasteur, Paris, France; New York University School of Medicine, United States of America

## Abstract

**Background:**

Eosinophils are effector cells during parasitic infections and allergic responses. However, their contribution to innate immunity has been only recently unravelled.

**Methodology/Principal Findings:**

Here we show that human eosinophils express CD3 and γδ T Cell Receptor (TCR) but not αβ TCR. Surface expression of γδTCR/CD3 is heterogeneous between eosinophil donors and inducible by mycobacterial ligands. Surface immunoprecipitation revealed expression of the full γδTCR/CD3 complex. Real-time PCR amplification for CD3, γ and δ TCR constant regions transcripts showed a significantly lower expression in eosinophils than in γδT cells. Limited TCR rearrangements occur in eosinophils as shown by spectratyping analysis of CDR3 length profiles and *in situ* hybridization. Release by eosinophils of Reactive Oxygen Species, granule proteins, Eosinophil Peroxidase and Eosinophil-Derived Neurotoxin and cytokines (IFN-γ and TNF-α) was observed following activation by γδTCR-specific agonists or by mycobacteria. These effects were inhibited by anti-γδTCR blocking antibodies and antagonists. Moreover, γδTCR/CD3 was involved in eosinophil cytotoxicity against tumor cells.

**Conclusions/Significance:**

Our results provide evidence that human eosinophils express a functional γδTCR/CD3 with similar, but not identical, characteristics to γδTCR from γδT cells. We propose that this receptor contributes to eosinophil innate responses against mycobacteria and tumors and may represent an additional link between lymphoid and myeloid lineages.

## Introduction

Eosinophils are polymorphonuclear granulocytes mainly found in increased numbers during helminth parasitic infections and allergic reactions [1], [2]. They are classically considered as mediator-releasing cells during effector phase of adaptive immunity, under the influence of T cell dependent cytokines or chemokines and antibodies [2], whereas eosinophil-derived chemokines have been recently shown to locally attract Th2 lymphocytes at lung inflammatory sites [3], [4]. Nevertheless, their precise function as beneficial or deleterious to the host still remains ambiguous, since highly toxic proteins present in eosinophil granules exert potent cytotoxic effects against non self targets such as parasites [5], [6] but also against stressed or necrotic host cells [7] and in asthma [8]. Eosinophils are foremost present in mucosal tissues in contact with the environment such as in gastro-intestinal tract and skin [2] and are characterized by their wide morphological and functional heterogeneity [9].

In addition to these effector functions, eosinophils produce several Th1, Th2 and regulatory cytokines, such as IL-10 [10], [11], which, in contrast to T cells, are stored within granules and promptly released upon activation [12]. Eosinophils also express MHCII and CD86 [10], [13], [14] and act as antigen-presenting cells [15]. Furthermore, eosinophils share with T cells expression of various receptors such as CD25 [16], [17], CD4 [18], CD28 [10], [14] and several members of the CD2 family, including 2B4 [19]. This wide array of molecules endows eosinophils with the ability to induce and regulate adaptive immunity.

However, the early appearance of eosinophils in agnathans, predating the appearance of the classical adaptive immune system [20] and the expression by eosinophils of several receptors involved in innate immunity, such as formyl peptide receptor [21], protease-activated receptors [22], [23] and TLR [24] further point toward a role for eosinophils in innate immunity. Eosinophils contribute to TLR-mediated immunity against viruses and mycobacteria [25], [26]. Indeed, we recently showed that TLR-2-dependent activation of human eosinophils induced α-defensin and ECP release and decreased mycobacteria growth [24]. Furthermore, expulsion of mitochondrial DNA by eosinophils has been shown to contribute to innate immune defences against bacteria [27]. Finally, eosinophil-tumor cell interactions and IL-4-dependent tumoricidal activity of eosinophils have been reported [28], [29]. Thus eosinophils appear functionally located at the interface between innate and adaptive immunity.

Strikingly, γδT cells are ancestral to other lymphoid populations such as αβT cells and B cells, they participate to both innate and adaptive immune responses, have a preferential mucosal localisation and might act as professional antigen-presenting cells [30] recognizing non-peptide antigens found on several pathogens, including mycobacteria, necrotic and tumor cells [31], [32].

These surprising similarities between γδT cells and eosinophils prompted us to investigate, whether, in addition to other T cell-associated receptors, human eosinophils expressed a γδT cell Receptor (TCR). We here report that human blood eosinophils express low levels of surface γδTCR/CD3, inducible by mycobacterial ligands. We show that eosinophils release granule proteins and cytokines upon activation by γδTCR agonists, including mycobacteria. Furthermore, we provide evidence that γδTCR contributes to eosinophil cytotoxic potential towards tumors.

## Results

### Human eosinophils express CD3 and γδTCR but not αβTCR

In order to investigate expression by human blood eosinophils of T cell associated receptors, we purified eosinophils by negative selection using magnetic beads. These highly purified eosinophils (Figure S1A) expressed specific granule proteins such as eosinophil peroxidase (EPO) and major basic protein (MBP) but not myeloperoxidase (MPO) associated to neutrophil and monocyte/macrophage lineages [33] (Figure S1B). Lymphocytes expressed neither of these myeloid markers (Figure S1B).

**Figure 1:**
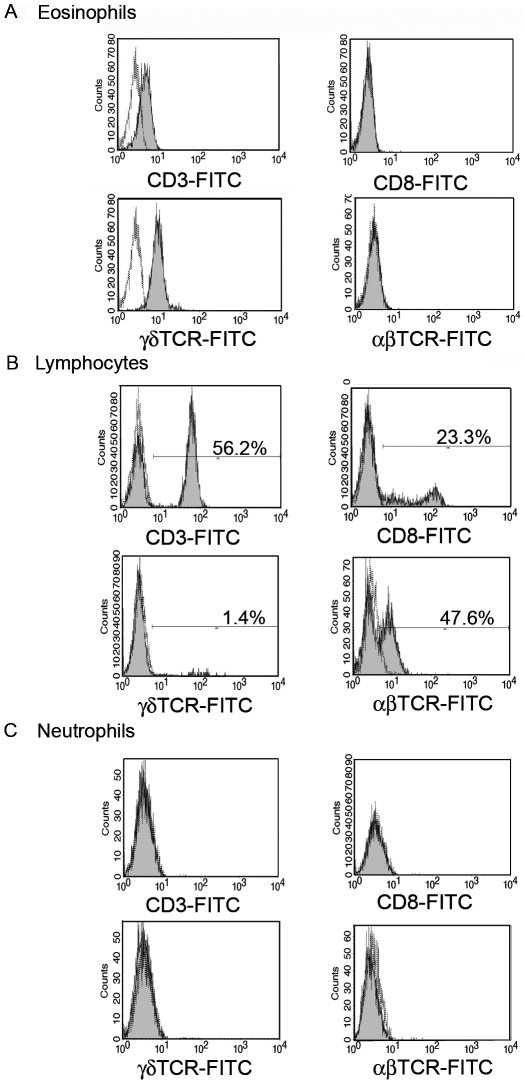
Expression of γδTCR/CD3 complex by human eosinophils. (A–C) CD3, CD8, αβTCR and γδTCR expression on permeabilized cells gated as described in Figure S1 (A) purified peripheral blood eosinophils (B) lymphocytes and (C) neutrophils. Staining with control isotype matched antibodies is represented in white histograms.

Following permeabilization, binding of anti-CD3 but not anti-CD8 antibodies was detected in eosinophils (Figure 1A). In T cells, CD3 associates with either αβTCR or γδTCR. We did not detect αβTCR on eosinophils but γδTCR expression was evidenced (Figure 1A). While lymphocytes from PBMC fraction expressed these markers at the expected frequencies (Figure 1B) and unlike a previous report [34], we were unable to detect CD3 or αβTCR expression in neutrophils (Figure 1C). Likewise neither CD8 nor γδTCR expression was detected in neutrophils (Figure 1C).

**Figure 2:**
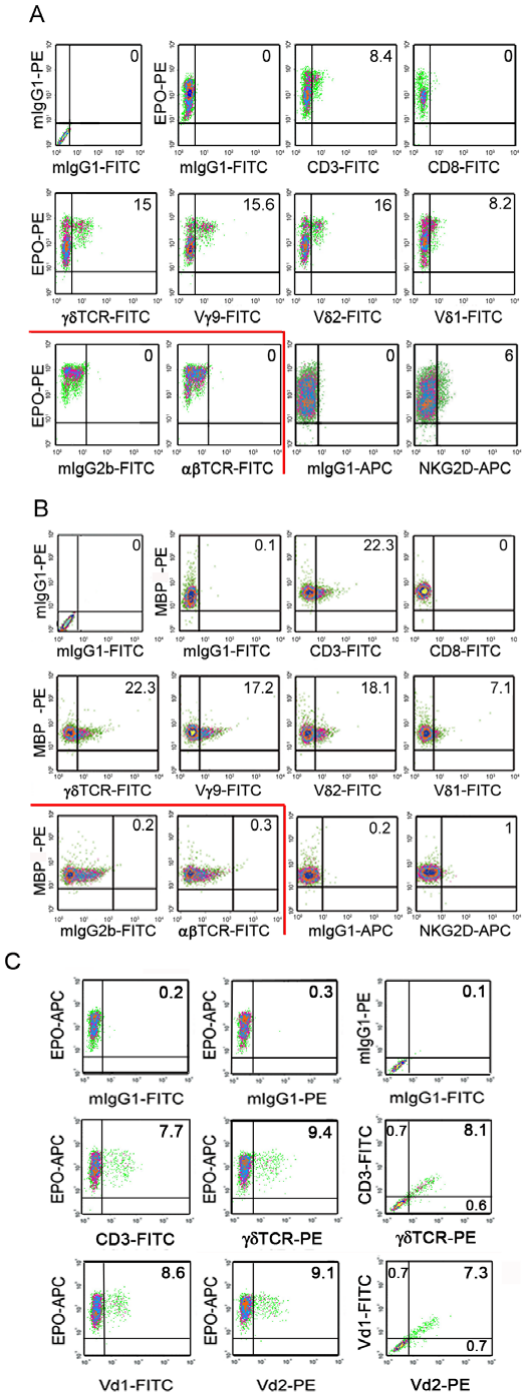
Surface expression of γδTCR/CD3 complex by human eosinophils. (A–C) CD3, γδTCR, Vγ9, Vδ2, Vδ1, αβTCR, CD8 and NKG2D surface expression on EPO^+^ (A) and MBP2^+^ (B) purified peripheral blood eosinophils analysed after 18 h culture and gated as described in Figure S1. Results in (A) and (B) represent to 2 distinct representative donors. Staining with control isotype antibodies is represented. (C) Presence of CD3^+^γδTCR^+^EPO^+^ and Vδ1^+^δ2^+^EPO^+^ eosinophils identified by triple staining. Staining with control isotype matched antibodies as well as corresponding double stainings is represented.

Surface expression of CD3 and γδTCR was detected on a fraction of eosinophils following double staining with antibodies against two specific eosinophil granule proteins EPO (Figure 2A) or MBP (Figure 2B). Similarly to intracellular staining, neither CD8 nor αβTCR were detected at eosinophil surface. By contrast, another γδT cell marker, NKG2D, was expressed in a lower proportion of cells (Figure 2A–B). As CD3 is required for receptor complex surface expression, CD3^+^γδTCR^+^ eosinophils were thus identified following triple staining (Figure 2C). In human blood, γδT cells express either Vγ9/Vδ2 or Vδ1 variable regions. We also evidenced that EPO^+^ or MBP^+^ eosinophils expressed Vγ9, Vδ1 and Vδ2 and unexpectedly co-expressed Vδ1 and Vδ2 at their surface (Figure 2A–C).

**Figure 3:**
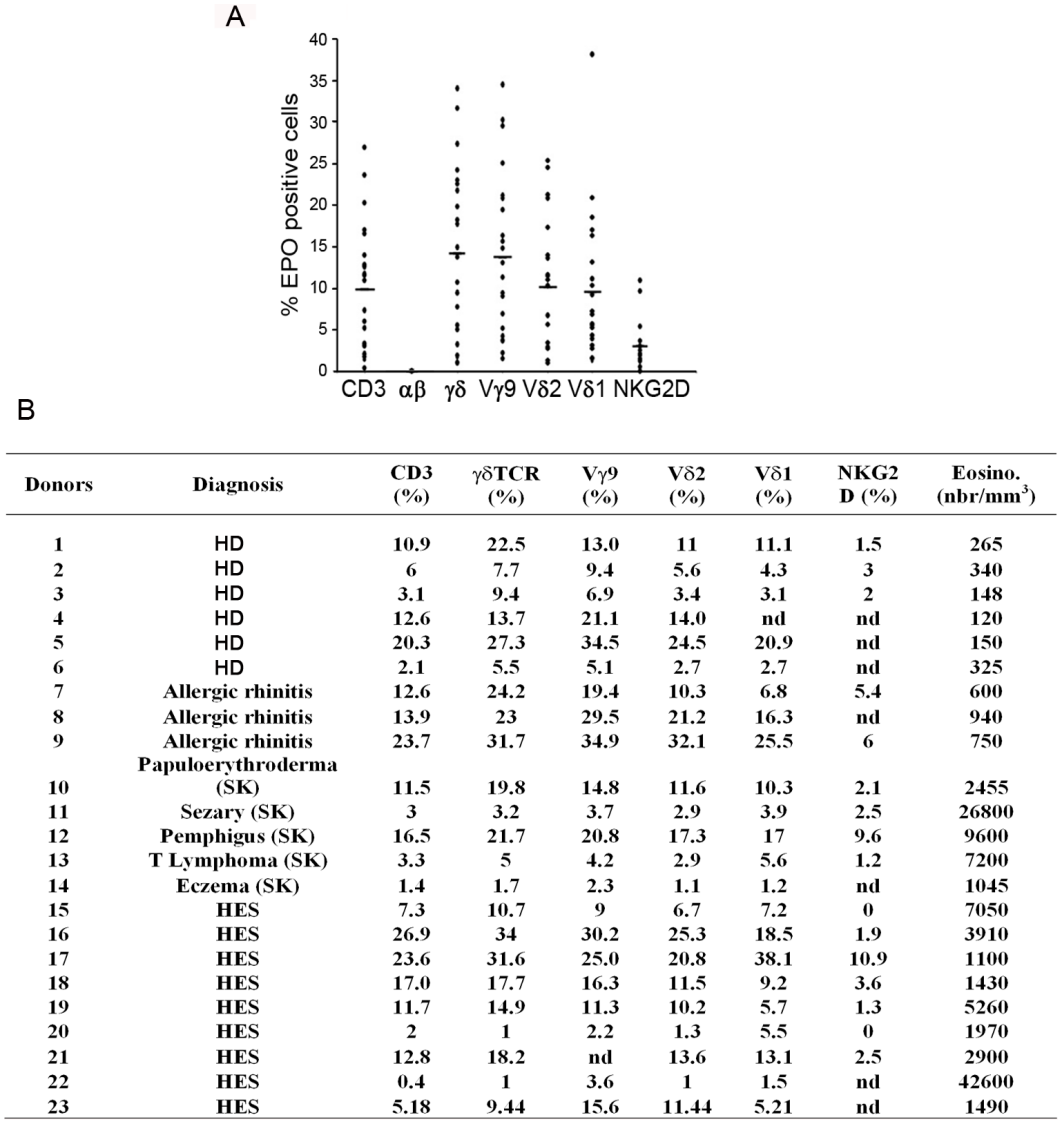
Heterogeneity of surface expression of γδTCR/CD3 complex by human eosinophils. (A) Cell surface marker expression on EPO^+^ gated peripheral blood eosinophils from 23 individual eosinophil donors. Group average expression for the indicated markers is represented (-). (B) Individual data and patient status.

Expression of γδTCR/CD3 complex was investigated in healthy donors and patients with either reactive eosinophilia (allergies, various skin diseases) or hypereosinophilic syndrome (HES). A wide heterogeneity of surface expression of γδTCR/CD3 and NKG2D was not only found among the 23 donors but also within each group, including healthy donors, however no correlation was observed between receptor expression levels and either eosinophilia, or a specific pathology (Figure 3A–B).

Following *in vitro* activation of γδT cells, CD3, CD8, γδTCR as well as Vγ9, Vδ2 and Vδ1 were detected at the expected frequencies (Figure S2A). Likewise, freshly isolated PBMC from the same eosinophil donors, in which γδT cells represent a very minor population, expressed CD3, αβTCR or low levels of γδTCR (Figure S2B). By contrast, monocytes did neither express CD3 nor γδTCR (Figure S2C). Finally, neutrophils neither expressed CD3, γδTCR nor the full αβTCR complex at their surface (Figure S2D). Thus, following overnight culture, a fraction of eosinophils, unambiguously identified by the presence of specific eosinophil markers, express the γδTCR/CD3 complex at their surface.

Besides receptor reexpression at the membrane following a resting period, as demonstrated for NKG2A on γδT cells [35], ligand-dependent induction or upregulation of receptor surface expression has been widely reported, including in the case of FcεRI [9]. Thus, we investigated whether a specific γδTCR ligand was able to induce γδTCR/CD3 surface expression on human eosinophils. While CD3 and γδTCR were barely detected at the surface of freshly purified eosinophils (Figure S3A), upon incubation for 2 h with TUBag1, a natural non peptidic ligand from *Mycobacterium tuberculosis* [36], about 50% of eosinophils expressed CD3 and γδTCR at their surface (Figure S3B). By contrast, neither CD8 nor αβTCR expression was induced upon incubation with TUBag (Figure S3B).

Finally, besides mature eosinophils present in peripheral blood, eosinophils can be differentiated *in vitro* from CD34^+^ cord blood progenitors. In such EPO^+^ eosinophils, in the absence of possible contamination by γδT cells, γδTCR/CD3 complex was detected at cell surface, after 3 weeks differentiation (Figure S4). As for blood eosinophils, neither CD8 nor αβTCR (not shown) could be detected.

### Eosinophils express all the subunits from γδTCR/CD3 complex

**Figure 4:**
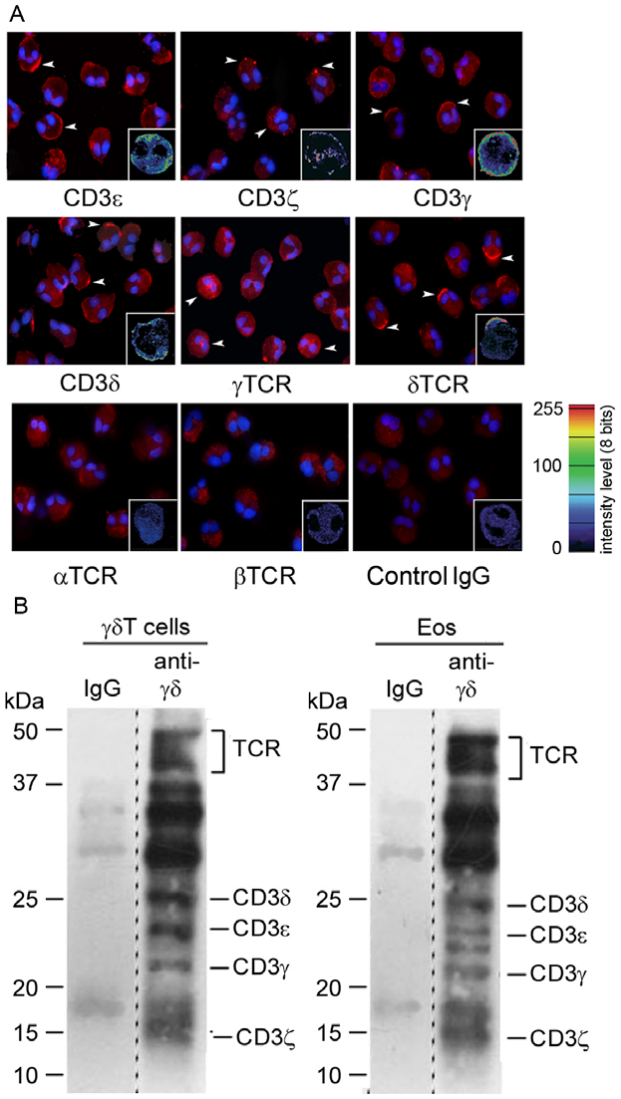
Immunolocalization and Subunit composition of surface-expressed γδTCR/CD3 complex on human eosinophils. (A) Immunofluorescence and confocal microscopy (insets) analysis of γδTCR/CD3 chains expression on cytospin eosinophil preparations after 18 h culture. Staining with anti-αTCR, anti-βTCR antibodies and control goat IgG is represented. Arrowheads indicate some positive cells. (B) Surface of eosinophils and γδT cells was biotin-labelled. Cells were lysed and complexes were immunoprecipitated, using anti-γδTCR or isotype control antibodies. Immunoprecipitated proteins were resolved on a reducing 14% SDS-PAGE and transferred to PVDF membrane. Biotinylated proteins were revealed using ABC-HRP and chemiluminescence. Positions of the TCR and CD3 subunits are marked. Material corresponding to the total and to 1/10^th^ of the material was loaded for eosinophils and γδT cells respectively. Dashed lines indicate that non adjacent lanes of the same gel have been joined on the Figure.

To evidence the cellular distribution of γδTCR/CD3 complex and to further exclude that results obtained by flow cytometry could be due to contaminating γδT cells, blood eosinophils were analysed at low magnification by immunofluorescence and at high magnification by confocal microscopy. In full agreement with flow cytometry data, in a preparation without contaminating cells, only a fraction of blood eosinophils, identified by their typical binucleated morphology, expressed the four chains of CD3 and γδTCR. No signal was detected with anti-αTCR, anti-βTCR antibodies or control IgG (Fig 4A). We next characterized the structure of the γδTCR/CD3 complex expressed at the surface of eosinophils, using surface biotinylation, followed by lysis with a mild detergent, co-immunoprecipitation with an anti-γδTCR antibody, SDS-PAGE in reducing conditions and detection of biotinylated proteins. Bands corresponding to TCR, CD3δ, ε, γ and ζ subunits were detected at the expected molecular weights both on γδT cells and eosinophils (Figure 4B). This result indicates that all the necessary chains are present to allow for surface expression of a complete γδTCR/CD3. However, the amount of material used for eosinophils was 10 times higher than for γδT cells, results consistent with flow cytometry data showing much lower surface expression in eosinophils than in γδT cells (Figure 2 and Figure S2).

### γδTCR/CD3 is less abundant and more restricted in eosinophils than in γδT cells

**Figure 5:**
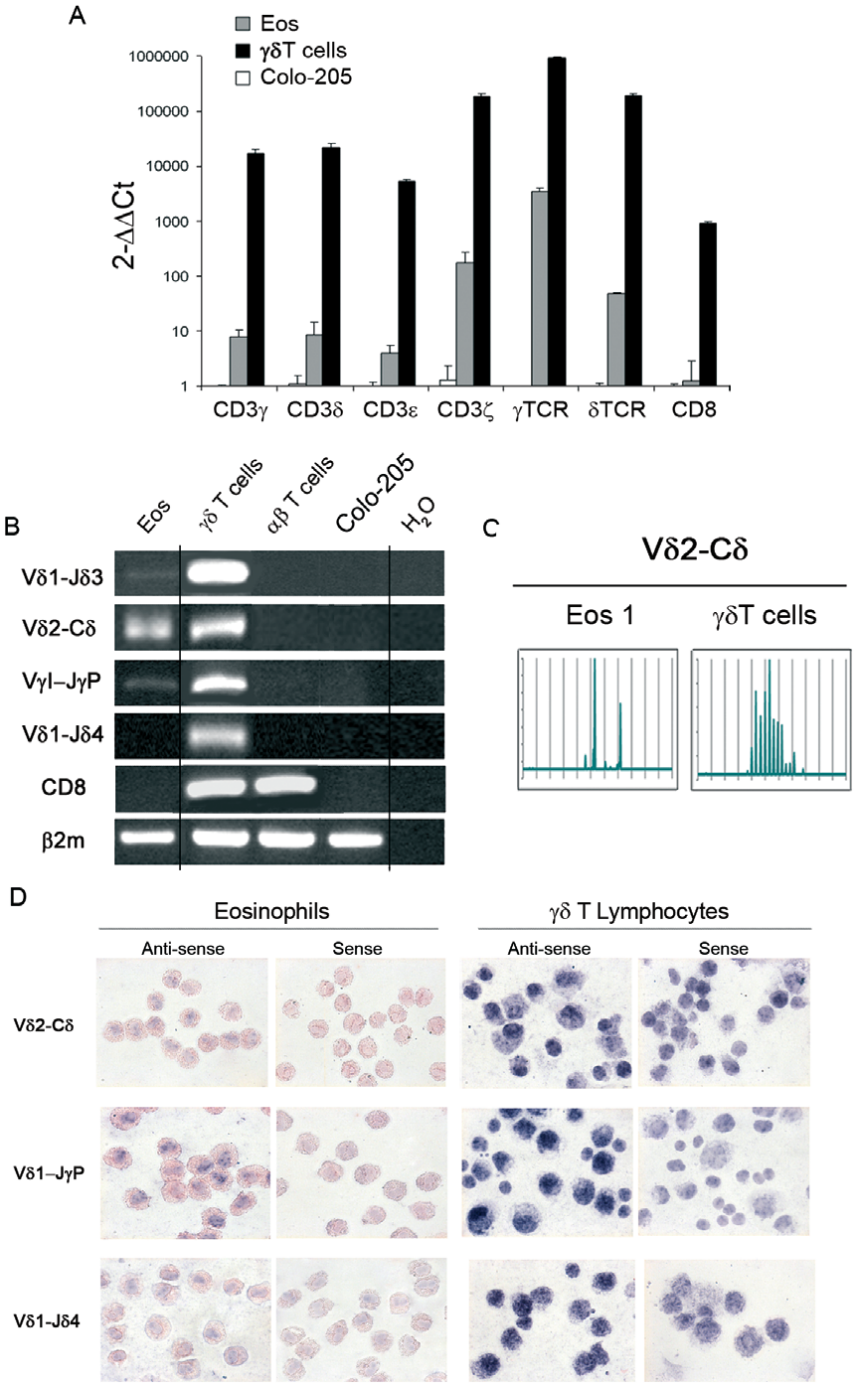
γδTCR/CD3 complex expression and rearrangements in human peripheral blood eosinophils. (A) Real time PCR analysis of expression of individual chains from γδTCR/CD3 complex in eosinophils, γδ T cells and Colo-205 cells. Average expression in Colo-205 cells is chosen as reference (samples were analysed in triplicate using primers listed in Table S1). (B) TCRγ and δ rearrangements in peripheral blood eosinophils analysed by RT-PCR using primers listed in Table S2. CD8 amplification is performed to exclude lymphocyte contamination. Colo-205 are chosen as negative control (C) Spectratyping analysis of CDR3 length profiles for Vγ9-Jγ1/2 ; Vγ9-JγP and Vδ2-Cδ families in eosinophils and in γδ T cells from the same donor. (D) Expression of rearranged γ and δTCR at the single cell level in peripheral blood eosinophils and γδT cells detected by *in situ* hybridization using anti-sense and control sense probes specific for Vδ2-Cδ, VγI-JγP and for Vδ1-Jδ4 used as negative control.

Transcripts specific for the four chains (ε, ζ, γ and δ) of CD3 complex, γTCR and δTCR constant regions and CD8 were amplified and quantified by real-time PCR in eosinophils purified to homogeneity (100%) and γδT lymphocyte subsets or Colo-205 colon carcinoma cells as positive and negative controls respectively. Expression of the various chains was 300 to 4,000 fold lower in eosinophil population than in γδT lymphocyte population, while CD8 expression was absent in eosinophils but present in γδT lymphocytes (Figure 5A). Amplification of Vδ1-Jδ3, Vδ2-Cδ and VγI-JγP rearranged transcripts but not Vδ1-Jδ4 was also detected in eosinophils. These rearrangements were all found in γδT lymphocytes but neither in αβT lymphocytes nor Colo-205 cells (Figure 5B). Spectratyping analysis of CDR3 length profiles for Vγ9-Jγ1/2, Vγ9-JγP and Vδ2-Cδ evidenced a limited Vδ2-Cδ diversity (2 peaks) on eosinophils compared to γδT cells (Figure 5C), while no signal was detected on eosinophils for the 2 other rearrangements, in contrast to γδT cells (data not shown). Furthermore, to unambiguously demonstrate that TCR rearrangements occur in eosinophils, at single cell level, we performed *in situ* hybridization using sequences from rearranged TCR genes as RNA probes. Hybridization with anti-sense probes corresponding to Vδ2-Cδ and VγI-JγP rearrangements, that were detected by RT-PCR, gave a positive signal on nuclei from eosinophils at times clearly bilobed. In keeping with RT-PCR results, a significantly stronger signal was detected on γδ T lymphocytes, while corresponding sense probes only gave background amplification on both cell types (Fig. 5D). Vδ1-Jδ4 anti-sense probe gave positive signal on γδ T lymphocytes but not on eosinophils in which this rearrangement was not detected by RT-PCR.

**Figure 6:**
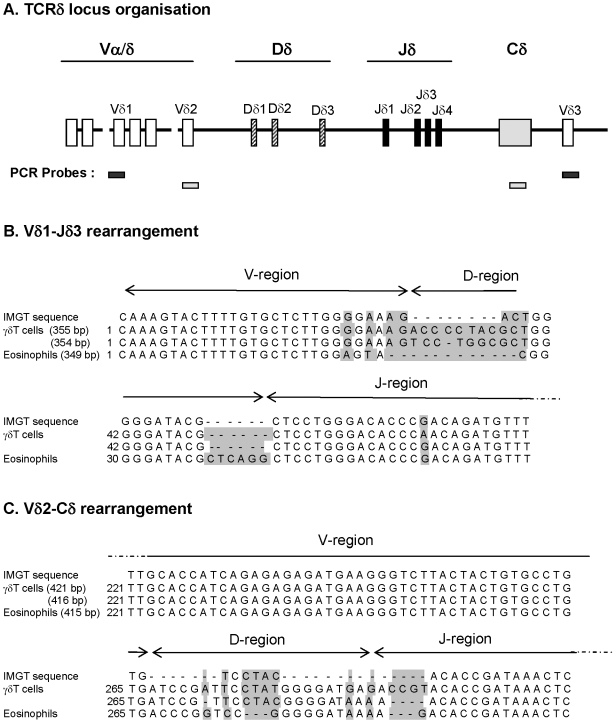
Intraindividual variability between eosinophils and γδT cells in rearranged Vδ1-Jδ3 and Vδ2-Cδ TCR sequences. (A) TCR locus organization with positions of the PCR probes used for sequence amplification. (B–C) Sequence alignment of Vδ1-Jδ3 (B) and Vδ2-Cδ (C) regions analyzed from IGMT database, differentiated γδT cells and peripheral blood eosinophils obtained from the same allergic donors for. The two most frequent sequences are presented for γδT cells. Grey boxes indicate mismatch.

Finally, DNA sequencing of Vδ1Jδ3 Vδ2-Cδ regions from eosinophils and γδT cells show that V and J regions were virtually identical to the IMGT sequence, while significant differences between eosinophils and 2 different cloned sequences of γδT cells from the same donor were found in D regions and in junctional regions (Figure 6). This further confirms that γδTCR/CD3 complex expressed by eosinophils is less expressed and diverse than the corresponding receptor on γδ T cells.

### γδTCR/CD3-mediated eosinophil activation induces ROS production, degranulation and cytokine release

**Figure 7:**
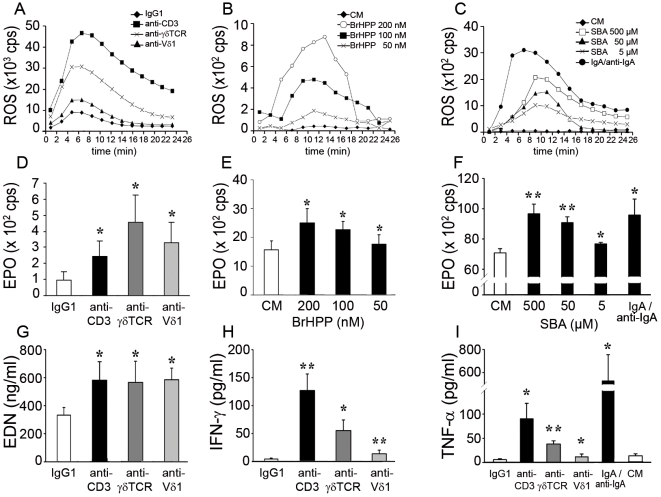
Specific mediator release upon CD3- or γδTCR-induced eosinophil activation. (A–C) Representative time course of ROS production following eosinophil activation by immunobilized specific antibodies (A), BrHPP (B) or SBA stimulation (C). (A) Stimulation with immobilized anti-CD3 (▪), anti-γδTCR (X) or anti-Vδ1 (▴) mAbs or control IgG_1_ (♦). (B) Stimulation by BrHPP 200 nM (○) 100 nM (▪) and 50 nM (◊) or culture medium (♦). (C) Stimulation by SBA 500 µM (□) 50 µM (▴) and 5 µM (X), culture medium (♦) or IgA/anti-IgA immune complexes (•). (D–F) EPO release by eosinophils upon CD3 or γδTCR activation by specific antibodies (D) (n = 6), BrHPP (E) (n = 3) or SBA stimulation (F) (n = 3). Results are expressed in count per second (cps). (G) EDN release by eosinophils upon CD3 or γδTCR activation by specific antibodies (n = 6). (H–I) Cytokine production by eosinophils upon CD3 or γδTCR activation by specific antibodies or IgA/anti-IgA immune complexes. IFN-γ production (H) (n = 5–7) and TNF-α production (I) (n = 4–8). Results presented in panels E and F were obtained on different patients. *, P<0.05; **, P<0.01.

Upon activation, eosinophils very rapidly produce Reactive Oxygen Species (ROS) and release, by selective degranulation, eosinophil-specific granule proteins, including highly cytotoxic cationic proteins such as EPO and eosinophil cationic protein (ECP) [6]. In purified blood eosinophils, γδTCR/CD3 complex activation by immobilized antibodies to CD3, γδTCR or Vδ1 led to a similar kinetics of ROS production (Figure 7A) and to a significant EPO and eosinophil-derived neurotoxin (EDN) release (Figure 7D and G). Stimulation with a soluble ligand, bromohydrin pyrophosphate (BrHPP), a phosphoantigen agonist selective for human γ9δ2^+^ T lymphocytes [37], induced dose-dependent ROS production as well as EPO release (Figure 7B and E). Furthermore, eosinophil activation by sec-butylamine (SBA) [38], an inducer of endogenous phosphoantigens, yielded to a sustained dose-dependent ROS and EPO production (Figure 7C and F), comparable to values obtained following activation with IgA/anti-IgA immune complexes, one potent physiological eosinophil activator. Of note, plate-bound antibodies induced stronger ROS release but weaker EPO release than soluble γδTCR activators. Since eosinophils also release several immuno-regulatory and proinflammatory cytokines [10], we further show that activation with anti-CD3, -γδTCR or -Vδ1 antibodies efficiently induced IFN-γ and TNF-α production by eosinophils (Figure 7H and I). Thus, γδTCR/CD3-expressing eosinophils respond to selective agonists similarly to human γδT lymphocytes and produce both specific eosinophil granule proteins, myeloid cell mediators as well as cytokines.

### *Mycobacterium bovis* induce γδTCR/CD3-dependent eosinophil activation

**Figure 8:**
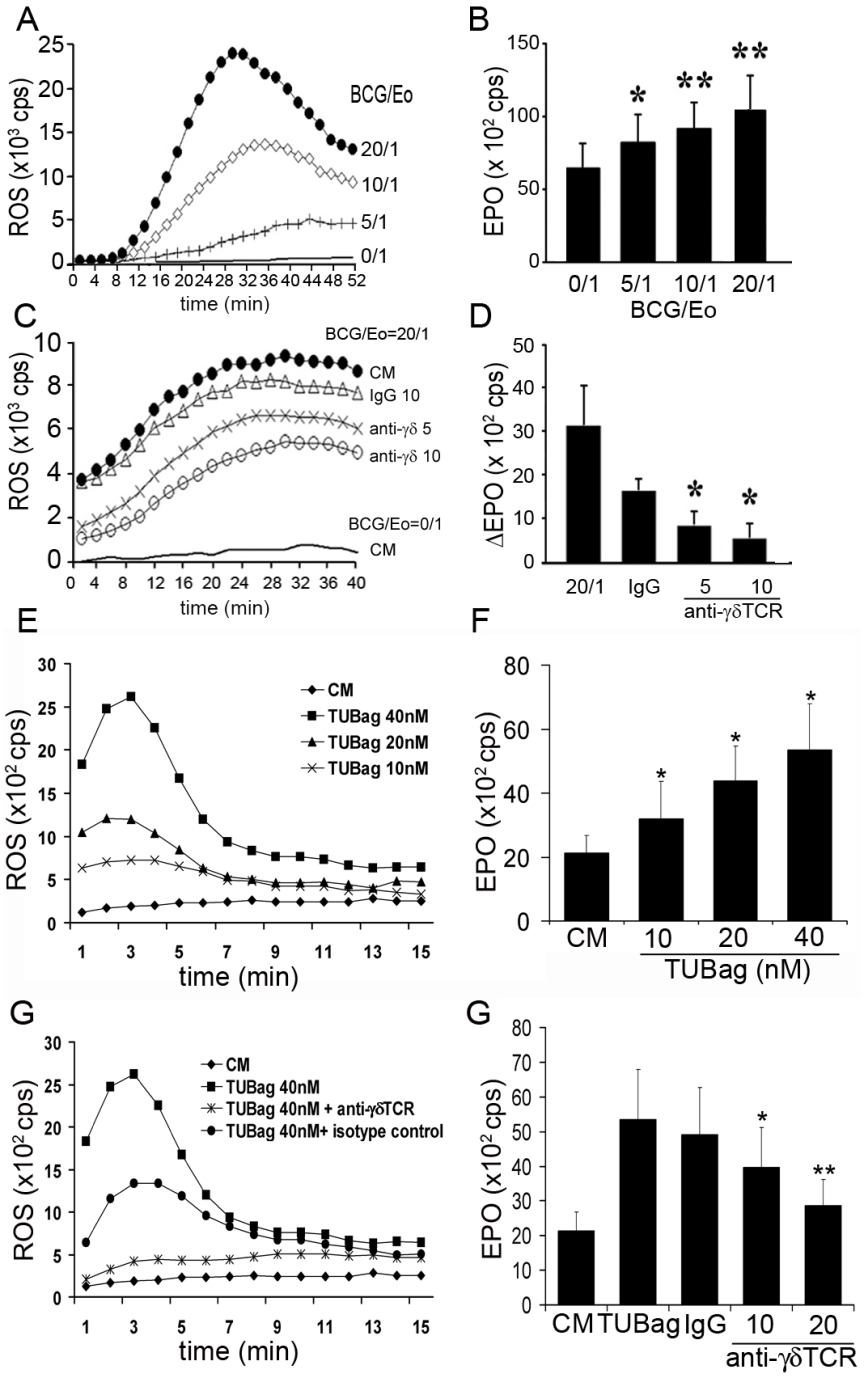
*Mycobacterium bovis*-BCG-induced eosinophil activation. (A and E) Dose-dependent ROS production by BCG- (A) or TUBag (E) activated eosinophils. BCG/eosinophil ratio: 20/1 (•), 10/1 (◊), 5/1 (+) and 0/1 (−). (B and F) Dose-dependent EPO release by eosinophils activated by different BCG/eosinophil ratios (n = 3) (B) or TUBag concentrations (F). (C and G) Inhibition of BCG- (ratio 20/1 •) (C) or TUBag- (G) induced ROS production by an anti-γδTCR blocking antibody 5 µg/ml (x) and 10 µg/ml (○) or an isotype control antibody (Δ). (D and G) Inhibition of BCG- (D) or TUBag- (G) induced EPO production by an anti-γδTCR blocking antibody (5 and 10 µg/ml) or an isotype control antibody. Results are expressed as ΔEPO cps values (values from medium stimulation are subtracted from values obtained with BCG stimulation for each antibody) (n = 3). (E–F) *, P<0.05; **, P<0.01.

Human γδT cells respond to non-peptide antigens including mycobacterial and tumor-derived ligands. Patients with mycobacterial infections may exhibit blood and tissue eosinophilia and mycobacteria-induced eosinophil-associated experimental acute inflammation [39] as well as rapid eosinophil chemotaxis *in vivo*. Mycobacteria- eosinophil interactions might thus reflect the reactivity conferred by γδTCR/CD3 complex. Accordingly, incubation of blood eosinophils with increasing ratios of *Mycobacterium bovis*-BCG induced dose-dependent ROS production and EPO release (Figure 8A–B), that could be inhibited, in a dose-dependent manner, by an anti-γδTCR antibody [40] (Figure 8C–D). Similar eosinophil activation, and inhibition by an anti-γδTCR antibody, was also obtained following incubation with TUBag, a natural mycobacterial component (Figure 8E–H). Thus mycobacteria appear capable of activating eosinophils, at least partly, in a γδTCR-dependent manner.

### γδTCR/CD3-dependent eosinophil cytotoxicity toward tumor cells

**Figure 9:**
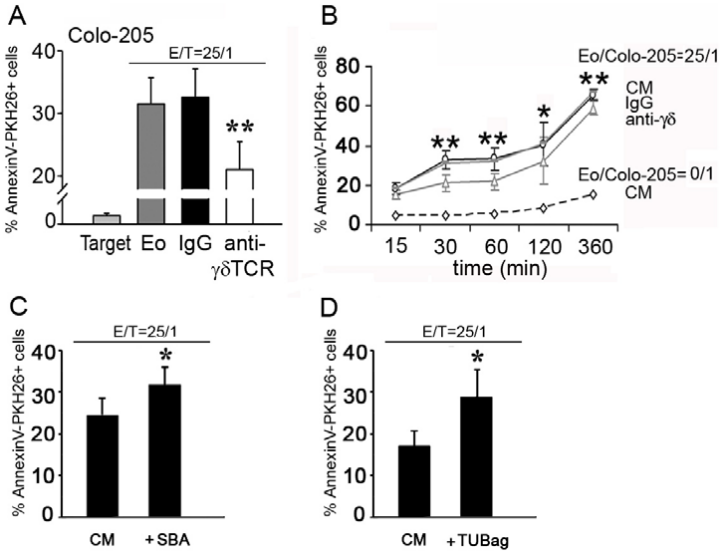
γδTCR-dependent tumor-cell killing by human eosinophils. (A–B) Induction of Colo205 killing by eosinophils and inhibition by an anti-γδTCR blocking antibody. Percentage of AnnexinV^+^ tumor cells in the presence or absence of eosinophils and blocking anti-γδTCR or isotype control antibodies (n = 5–7) at various time-points. Panel A represents the 60 min time-point. (C–D) Potentiation of eosinophil-mediated Colo205 killing by γδTCR ligands. (C) 50 µM SBA (n = 7). (D) 20 mM TUBag (n = 4). *, P<0.05; **, P<0.01.

Eosinophils have been associated with many tumors *in vivo*. Furthermore, both γδ T cells [41] and eosinophils [19] exert potent cytotoxic activity towards many tumor cells. Therefore, we investigated γδTCR-mediated eosinophil cytotoxicity towards Colo-205 carcinoma cell line. Indeed, eosinophils induced time-dependent apoptosis of Colo-205 tumor cells *in vitro*, which was significantly inhibited following addition of neutralizing anti-γδTCR Ab (Figure 9A). This effect was more prominent at earlier time-points suggesting that γδTCR-mediated eosinophil-tumor interactions are important for the initiation of the cytotoxic reaction (Figure 9B). Finally preincubation of eosinophils with γδTCR ligands, SBA and TUBag, potentiated eosinophil cytotoxicity towards tumor cells (Figure 9C–D). Altogether, these data confirm the potential role of eosinophils in anti-tumor cytotoxicity and strongly suggest the involvement of γδTCR/CD3 complex in this mechanism.

## Discussion

We here provide evidence that highly purified human blood eosinophils, as well as eosinophils differentiated *in vitro* from CD34^+^ cord blood progenitors, gated on the basis of presence of specific eosinophil granule proteins, express a functional γδTCR/CD3 complex, so far almost exclusively associated to T lymphocytes, but lack αβTCR. As previously observed for other receptors such as CD25 [42], CD4 [18], FcεRI, CD89 and CD28 [10], γδTCR/CD3 surface expression on eosinophils is highly heterogeneous between individual donors. Such an heterogeneity in γδTCR/CD3 expression has also been observed for human blood γδT lymphocytes [35]. As it is the case for several receptors, including FcεRI [9], surface expression of γδTCR/CD3 can be induced/up-regulated, in particular upon incubation with ligands. Our results show that γδTCR surface expression was induced upon incubation in the presence of a natural mycobacterial ligand as well as following overnight culture. The molecular mechanism underlying this later finding remains unknown. Our results indicate that CD3ε expression, both at mRNA and protein levels, was consistently lower than γδTCR. Due to the requirement of this CD3 subunit for surface expression of the receptor complex, it is tempting to speculate that the very low CD3ε expression probably accounts for the low surface expression of the whole complex on eosinophils although further experiments would be required for a formal demonstration. This low receptor expression as well as the fragility of eosinophils was a constraint to obtain significant amounts of viable γδTCR^+^ eosinophils following cell sorting.

Eosinophils display a rearranged γδTCR with a more restricted repertoire compared to γδT cells, further excluding the possibility of lymphocyte contaminants in eosinophil preparations. Besides T lymphocytes, δTCR rearrangements have only been reported in malignant B lymphoblasts [43], NK cells [44] and in mixed-type leukaemia retaining both T-lymphoid and myeloid characteristics. Furthermore, presence of double positive Vδ1^+^Vδ2^+^ and equal numbers of Vδ1- and Vδ2-expressing eosinophils in most of normal donors suggests incomplete allelic exclusion as found in some instances in γδT cells [45]. Direct γδTCR activation of eosinophils by γδTCR/CD3-specific antibodies and selective agonists induced eosinophil-specific responses. Release of such myeloid-associated mediators and the scarcity of circulating γδ T cells further exclude the possibility that our results obtained using highly purified eosinophils would be due to γδTCR expression by contaminating lymphocytes.

Thus, mature eosinophils expressing lymphoid markers might represent hitherto unattended γδTCR-expressing myeloid cells. Our results might be in agreement with observations that myeloid-derived plasmacytoid DC are able to express a “lymphoid program” (RAG1, rearranged IgH genes) [46]. By contrast, in the recently proposed myeloid-based model of haematopoiesis, T- or B-committed progenitors would each keep a myeloid-differentiation potential [47]. For instance, genetic reprogramming from differentiated B cells to macrophages has been reported [48]. Further experiments are required to unravel by which mechanisms eosinophils have acquired this lymphoid program. Of note, eosinophils are found in human thymus [8]. Furthermore, similarly to gut intraepithelial γδT cells, eosinophils are able to undergo peripheral differentiation, for instance in allergic lungs [49]. Investigating TCR expression by eosinophils in various organs and tissues would greatly benefit from experiments on animal models. Unfortunately, we were unable to detect γδTCR/CD3 on mouse blood and tissue eosinophils. This is not extremely surprising since mouse eosinophils are strikingly different from human eosinophils, regarding their granular content [50] and membrane expression of various immune receptors, in particular IgE receptors [51] and IgA receptors [52]. Furthermore, γδT cells also display significant differences between mouse and humans. Indeed, a high proportion of skin lymphocytes express γδTCR in mouse but not in human [53], while only human Vγ9Vδ2 are responsive to phosphoantigens [54].

Our findings indicate that eosinophils might also contribute, through γδTCR-dependent mechanisms, to immune defences in tissues, where 90% eosinophils are located. One might speculate that γδTCR/CD3 expression is higher in tissue than in blood eosinophils, as previously reported for FcεRI in a model of “humanized” mice where human FcεRI was quantified and increasingly expressed from blood to peritoneal cavity and spleen eosinophils [9]. Tissue eosinophils are associated to granulomatous diseases such schistosomiasis [55], Crohn's disease [56] and mycobacterial infections [39]. Eosinophils can damage cell wall and lyse *Mycobacterium tuberculosis in vitro* [57]. Vγ9δ2 and Vδ1 expression, rapid response to phosphoantigen ligands, as well as BCG-induced activation so far represented hallmarks of γδT lymphocyte-mediated cytotoxicity towards phosphoantigen-expressing mycobacteria [58]. Since tumors produce phosphoantigens and are targeted by γδT lymphocytes [58], γδTCR-mediated activation might thus contribute to the anti-tumoral activity of eosinophils. Indeed, while γδT lymphocytes and NK cells display granzyme B/perforin-mediated cytotoxicity, eosinophils additionally possess a unique set of cationic proteins with a strong cytotoxic potential [50]. Furthermore, tumor-associated tissue eosinophilia (TATE) is considered by some authors as a marker of good prognosis, including in colon carcinoma [59]. Anti-tumoral activity of eosinophils should be considered in the context of current development of innovative cancer immunotherapies, based on synthetic phosphoantigens [60]. Indeed, due to their high cytotoxic potential and despite their low γδTCR expression in comparison to T cells, eosinophils might thus represent very efficient “innate” effector cells in defence against targets bearing γδTCR ligands, such as tumor cells, in particular when eosinophils surround or infiltrate solid tumors.

Our work outlines a previously unexpected link between eosinophils and γδT lymphocytes, which not only share the expression of the γδTCR/CD3 complex but also some major functions in innate immunity. It might also shed light on the molecular mechanisms of differentiation, diversity and function of T-cell expressed γδTCR.

## Materials and Methods

### Ethics statement

Peripheral venous blood was obtained from healthy donors or eosinophilic patients after written informed consent. Research protocol was approved by the Comité Consultatif de Protection des Personnes dans la Recherche Biomédicale de Lille (France).

### Cell purification and culture

Eosinophils were isolated as described [61] on Percoll followed by negative selection using anti-CD16-coated microbeads. Purity determined after cytospin and RAL555 staining was above 98% and 100% for RNA analyses. Purified eosinophils were cultured overnight at 37°C in complete RPMI without phenol red.

Neutrophils were obtained by positive selection with anti-CD16 coated microbeads. Purity was 99%. For RNA extraction, αβTCR lymphocytes from PBMC from normal donors were sorted after staining with anti-αβTCR-PE using FacsAria™ cell sorter and Diva™ software (BD). Purity was 99%. γδTCR lymphocytes were generated from PBMC (0.5×10^6^/ml) by culture in complete RPMI 1640 medium with 200 nM bromohydrin pyrophosphate BrHPP (Innate Pharma, Marseille) and 20 ng/ml rhIL-2 for 7 days. At that time, 50–70% cells were γ9δ2TCR^+^ cells. For RT-PCR and *in situ* hybridization, γδTCR^+^ cells were further purified using anti-γδTCR microbeads (Miltenyi).

Cord blood was obtained from the maternity unit of Lille University Hospital. Mononuclear cells were isolated by Ficoll-Hypaque density centrifugation. CD34^+^ cells were purified using CD34^+^ isolation kit (Miltenyi). Purified CD34^+^ cells were cultured in complete medium supplemented with 2.5×10^−5^ M β-mercaptoethanol (β-ME) at 0.5×10^6^ cells/ml. Eosinophil differentiation was induced upon addition of 50 ng/ml SCF, 1 ng/ml each GM-CSF, IL-3 and IL-5 (Peprotech).

Colo-205 colon carcinoma cell line was obtained from ATCC. Cells were maintained in RPMI 1640 medium supplemented with 10% FCS, 10 mM L-Glutamine and 10 µg/ml Gentamycin.

### Flow cytometry

Antibodies and isotype controls used for flow cytometry (origin, clone, working dilution or concentration) are listed in Table S1. Anti-EPO (AHE) and anti-MBP2 [62] (a kind gift from Dr. D Plager, Mayo Clinic, Rochester) were biotinylated using biotin-sulfosuccinimidyl ester (Molecular Probes) according to manufacturer's instructions and used at a 1∶100 dilution. Samples were analysed on a FACSCalibur™ using CellQuest™ software (BD). Multiple staining experiments are represented as color density plots [63] with linear scales used for both forward (FSC) and side scatter (SSC) and logarithmic scales used for fluorescence parameters (Log density: 50%).

Intracellular staining was performed after fixation of purified eosinophils, neutrophils or PBMC (2×10^5^) with 2% paraformaldehyde and permeabilization with 0.01% saponin in PBS. After blocking of non-specific binding with 5 µl mouse serum for 10 min, cells were incubated with anti-CD3-FITC, anti-αβTCR-FITC, anti-γδTCR-FITC or anti-CD8-FITC or corresponding isotype controls for 30 min in the presence of saponin.

Following incubation for 2 h with or without TUBag-1 [36] (40 nM) in RMPI 1640 or after overnight culture, purified blood eosinophils were gated prior to the analysis, on the basis of their FSC and SSC characteristics in order to exclude dead cells (Figure 1B). Within PBMC fraction, monocytes and lymphocytes were gated on the basis of their FSC and SSC characteristics prior to the analysis. Quadrants were set on samples after double staining with FITC- or APC- and PE- or biotin-conjugated control isotype antibodies. At least 10^4^ events were acquired per sample.

For double and triple staining, cultured peripheral eosinophils were preincubated with 5% human serum albumin (HSA) for 30 min at 4°C and labelled for membrane receptor with the relevant fluorochrome-conjugated antibodies. After washing, cells were fixed 10 min with 2% paraformaldehyde at 4°C and permeabilized for 10 min at room temperature (RT) with 0.01% saponin in PBS-1% BSA. Non-specific binding was blocked with 5 µl mouse serum for 10 min. Purified peripheral blood eosinophils were further identified by incubation with biotinylated anti-EPO or anti-MBP or isotype control for 30 min and streptavidin-PE (Molecular Probes) (1∶200) for 20 min in the presence of saponin. Neutrophils were identified by intracellular staining with anti-MPO-PE following an identical procedure. Monocytes and T lymphocytes were identified by staining with anti-CD14-PE, anti-αβTCR or anti-γδTCR respectively.

### Immunofluorescence and confocal microscopy

Purified eosinophils were cytocentrifuged, fixed in 4% paraformaldehyde, washed in 0.05 M PBS. Fixation was stopped with 0.1 M glycine for 4 min and 50 mM NH_4_Cl pH 7.4 for 15 min. After each incubation step, cytospins were washed for 10 min in PBS. Cells were incubated with PBS-3% BSA-5% HSA for 30 min and, after one PBS wash, with 10% decomplemented donkey serum (Jackson ImmunoResearch). Cytospins were then incubated overnight at 4°C in a humid chamber with goat antibodies specific for CD3ε, CD3ζ, CD3γ, CD3δ, αTCR, βTCR, γTCR, δTCR (Santa Cruz) or with goat IgG (Jackson ImmunoResearch) in PBS-BSA-HSA (40 µg/ml). After washing in PBS-0.1% BSA, cells were incubated with biotinylated donkey anti-goat IgG (Jackson ImmunoResearch) (1∶200) in PBS-BSA-HSA for 2 hours, followed by streptavidin-Texas Red (Molecular Probes) (1∶500) in PBS-3% BSA for 1 hour. Final washes were with PBS-0.1% BSA- 0.02% Tween 20 for 2×10 min and with PBS for 10 min. For immunofluorescence, DNA was stained with Hoechst 33342 (Invitrogen) (1∶1000), immediately prior to cytospin mounting in Fluoromount G (Southern Biotechnologies).

For conventional immunofluorescence a Leica DM-RB microscope equipped for epifluorescence with a 100 W mercury lamp was used. Filter and dichroic mirror sets were TX2 for Texas Red and A for Hoechst 33342. Images were acquired with a 100×1.32 NA oil immersion objective, a Photometrics Cool SNAP™ camera using RSImage™ software (Roper Scientific). Samples were analysed by confocal microscopy using a DM-IRE2 inverted microscope with SP2-AOBS scan-head (Leica) at the Imaging Facility of Institut Pasteur de Lille. Excitation was performed at 543 nm for Texas Red. Fluorescence emission wavelengths pass bands were selected between 581 and 621 nm according to the emission spectral analysis. Excitation power was between 100 and 400 µW. Acquisitions were performed using a 100×1.4 NA oil immersion objective. 3D pre-treatment, analysis and edition were performed with Edit3D (free software, Y. Usson, GDR2588, CNRS, France). Analysis and editing was performed on Photoshop (Adobe).

### Surface immunoprecipitation

Surface labelling and co-immunoprecipitation was performed as previously described for γδ T cells [64]. Purified human eosinophils or differenciated γδT cells were washed and then resuspended in PBS (pH 8.0) at 3.10^7^ cells/ml at room temperature. Surface biotinylation was performed for 30 min at room temperature using 2 mM Sulfo-NHS-LC-Biotin (Pierce Biotechnology). Cells were washed three times with PBS containing 100 nM glycine and once in PBS.

Labelled cells were lysed in a buffer containing 20 mM Tris (pH 7.5), 150 mM NaCl, 1 mM EDTA, protease inhibitors and 1% Brij 97 (Sigma-Aldrich). Lysates were cleared by centrifugation for 15 min at 12,000 *g* and sequentially incubated at 4°C in the presence of Protein-G Sepharose (Gamma bind Plus Sepharose, Pharmacia), Protein G Sepharose-bound normal mouse IgG (Jackson Immunoresearch) and Protein G Sepharose-bound anti-γδTCR. Immunoprecipitated samples were washed three times with lysis buffer then boiled in reducing sample buffer (30 mM Tris, pH 6.8, 5% glycerol, 2% SDS, 2.5% β-mercaptoethanol, 0.1% bromophenol blue). Material was run on 14% polyacrylamide gels. Material corresponding to the total and to 1/10^th^ of the material was loaded for eosinophils and γδT cells respectively. Following separation, proteins were transferred to PVDF membrane (Biorad). Blots were developed using ABC-HRP (Vector Laboratories) and the enhanced chemiluminescence (ECL) plus Western blot detection system (Amersham).

### PCR analysis

Total RNA was isolated using the RNeasy mini kit (Qiagen) from 10^7^ purified cells and reverse transcription was performed using SuperScript™RT (MMLV 200 U/µl) (Invitrogen). cDNA was amplified using primers (Proligo) (20 pmoles/µl) listed in Table S2. PCR were run for 40 cycles (1 min at 95°C, 1 min annealing and 1 min at 72°C) using Taq polymerase (Bioprobe).

### Real time PCR

Total RNA was prepared from eosinophils by guanidium/CsCl centrifugation method. Briefly, purified eosinophils were pelleted by centrifugation and then lysed in 4 M guanidium isothiocyanate, 1 mM EDTA, 25 mM sodium acetate, 4.9% β-mercaptoethanol, 68 mM N-lauryl sarcosine. Lysate was drawn through a 20G needle. RNA was obtained by ultracentrifugation (28000 rpm, 20 h, and 20°C) through a CsCl gradient. RNA pellet was washed twice and dissolved in water and precipitated overnight at −20°C with ethanol 70% and sodium acetate 0.08 M. After centrifugation (10000 rpm, 45 minutes at 4°C), RNA was washed twice in ethanol 70%, resuspended in water and store at −80°C until use. RNA from γδ T cells and Colo205 cells was prepared using RNeasy mini-spin columns (Qiagen, UK) according to the manufacturer's instructions. All samples were quantified by absorbance measurement at 260 nm on a spectrophotometer (Biorad), and RNA quality was checked by running samples on 1.5% agarose gel in RNA loading buffer (Sigma).

Total RNA was first submitted to DNAse I (Invitrogen) treatment (15 min at room temperature), and cDNA was generated using Superscript II reverse transcriptase (Invitrogen) according to the manufacturer instructions. Samples were analyzed by quantitative real-time PCR, performed according to manufacturer's protocol, using SYBR Green PCR Master Mix (Applied Biosystems) and the ABI Prism 7000 Sequence Detection System (Applied Biosystems). Primers were designed using the Primer3 Website and are listed on Table S2.

Samples were run in triplicate in a reaction volume of 25 µl. Amplification was carried out for 40 cycles, with denaturation at 95°C for 10 min during the first cycle and subsequently for 15 seconds, annealing and extension for 1 minute at 60°C. A dissociation temperature gradient was included at the end of the run. Gene expression was normalized according to GAPDH expression. Relative gene expression was calculated with the 2^−ΔΔC^T method [65].

### TCR repertoire analysis

Qualitative analysis of CDR3 length for Vγ9-Jγ1/2, Vγ9-JγP and Vδ2-Cδ families was performed in triplicate on eosinophils and in γδ T cells from the same donor (TcLand, Nantes, France) [66]. Briefly, cDNA was amplified by PCR using a Cγ or Cδ reverse primer and Vγ9 or Vδ2-specific forward primers, respectively. The amplifications were performed in a 384-Well GeneAmp® PCR System 9700 (Applied Biosystems, Foster City, CA). Briefly, each amplification product was used for an elongation reaction using a dye-labeled Jγ specific-labelled primer for the rearrangement analysis and Cδ labeled primer for the δ chain analysis, then heat denatured, loaded at least in duplicate onto a sequencing analyzer (48-capillary 3730 DNA Analyzer - Applied Biosystems). Then, GeneMapper software (Applied Biosystems) was used to display the distribution profiles of CDR3 lengths, in amino acids, of the amplified and elongated products.

### γδTCR junctional sequences analysis

Vδ1-Jδ3 and Vδ2-Cδ amplification products (400 bp) were cloned into a TA vector (pCR-TOPO, Invitrogen) using standard protocols. For each rearrangement, sequencing was performed on 5 randomly picked clones. TCR sequences were obtained from the IMGT website (imgt.cines.fr). Sequence alignments were performed using DNAstar software. GeneBank sequence accession numbers are: FJ890312 and FJ890313 respectively.

### *In situ* hybridisation

PCR products, amplified from purified γδTCR lymphocyte RNA, and corresponding to VγI-JγP, Vδ2-Cδ and Vδ1-Jδ4 rearrangements were cloned into the pCRII-TOPO vector (Invitrogen). Clones were isolated and sequenced on both strands. Clones corresponding to the published sequence were used to generate hybridization probes. Sense and anti-sense probes were synthesised from linearised plasmids using 350 µM digoxigenin-UTP (Roche) and SP6 or T7 polymerases as described [67]. *In situ* hybridisation was performed using a Discovery automat and corresponding kits (Ventana Medical Systems). Slides were incubated in EZprep buffer before processing with a standard RiboMap Kit. Slides were pre-treated 30 min with CC2 buffer, then 20 min with protease 3, followed by 6 h hybridization with sense or anti-sense probes (100 ng/slide). Slides were then washed twice 10 min in 2× SSC at 60°C, twice 10 min in 0.1× SSC at 60°C. Slides were incubated for 30 min with a mouse anti-digoxigenin antibody (Jackson ImmunoResearch) and then for 30 min with a rabbit anti-mouse antibody before treatment with UltraMap kit. Labelling was detected after a 150 min (Vδ2-Cδ), 110 min (VγI-JγP) or 90 min (Vδ1-Jδ4) incubation in the presence of NBT/BCIP substrate. Slides were counterstained for 1 h with Nuclear Fast Red and mounted in permanent mounting medium (Vector).

### ROS production and EPO release

ROS production and EPO release were measured as described [61]. Wells were coated with 10 µg/ml anti-mouse IgG F(ab')_2_ (Sigma) for 2 h at 37°C, followed by incubation with 10 µg/ml anti-CD3, anti-γδTCR, anti-Vδ1or isotype control mouse IgG_1_ for 2 h at 37°C. Eosinophils (2×10^6^/ml) were added in the presence of luminol (8.3 µg/ml). Alternatively, eosinophils were stimulated with 50–200 nM BrHPP, 5–50 µM sec-butylamine (SBA) (Sigma) or IgA/anti-IgA immune complexes (incubation with 7.5 µg/ml secretory IgA for 1 hr then by addition of 10 µg/ml anti-IgA). Chemiluminescence was measured at 37°C for 5 sec/timepoint with a luminometer (Victor^2^™ Wallac). For EPO release, supernatants (50 µl) were transferred into a microtiterplate and luminol (50 µg/ml), D-luciferin (4×10^−5^M) (Roche Diagnostics) and H_2_O_2_ were injected.

### EDN and cytokine release

Eosinophils (2×10^6^/ml) were activated with the same stimuli as for ROS production. After 18 h culture, supernatants were collected. EDN levels were measured by specific ELISA (MBL). The lower detection limit was 0.62 ng/ml. IFNγ and TNFα cytokines were measured by specific ELISA (Diaclone). Detection limit was 5 pg/ml for IFNγ and 8 pg/ml for TNFα.

### Activation with Mycobacteria

*Mycobacterium bovis*-BCG Pasteur strain was obtained from Dr C. Locht (Inserm U629, Institut Pasteur de Lille, France) and was grown in Sauton medium at 37°C. Eosinophils were incubated with various BCG numbers (Eosinophil/BCG: 20/1, 10/1 and 5/1). ROS and EPO release were determined as described above. For inhibition experiments, eosinophils (20/1 ratio) were incubated with a blocking anti-γδTCR mAb (10 µg/ml) or isotype control for 30 min at 37°C, before BCG addition.

### Tumor cell apoptosis

Colo 205 cells were labelled with 10 µM PKH26 (Sigma) according to manufacturer's instructions. Then, eosinophil-mediated cytotoxicity against PKH-26-labelled Colo-205 (10 µM) was measured in complete medium at 1.6×10^4^ targets/well into U-bottom plates containing eosinophils (25∶1 Effector:Target ratio) [61]. After different time points, apoptosis was measured by flow cytometry after annexinV-FITC staining (Pharmingen) for 15 min at RT. For inhibition experiments, blocking antibodies against γδTCR or isotype-matched control (10 µg/ml) were added to eosinophils immediately prior to the incubation in the presence of targets. In some experiments, eosinophils were preincubated for 30 min with 50 µM SBA or 20 mM TUBAg 1 before addition to the target cells.

### Statistical analysis

Individual data or mean±S.E.M values were presented. All statistical analyses were performed using SPSS software. Normality of data samples was assessed with the Normality test of Shapiro and Wilk. Then parametric paired-samples Student's t-test was used to compare variables and the one-tailed statistical significance level was as represented on Figures.

## Supporting Information

Figure S1 Gating strategy and specific identification of purified peripheral blood eosinophils in flow cytometry analysis. (A–B) Gating of purified peripheral blood eosinophils on forward and side scatter parameters immediately after purification. (A) Ellipsoids circle the analyzed population. (B). Specificity of eosinophil identification by intracellular detection of EPO, MBP or MPO using PE- or biotin-conjugated specific antibodies (grey shaded histograms). Staining on purified neutrophils as well as gated lymphocytes and monocytes from purified PBMC is shown as control. Staining with control isotype matched antibodies is represented in white histograms.
(6.41 MB TIF)

Figure S2 Surface expression of CD3, γδTCR, Vgamma9, Vdelta2,Vdelta1, αβTCR, CD8 and NKG2D detected by flow cytometry in double staining on immune cell populations. (A) In vitro-generated BrHPP-induced gamma9 delta2 TCR^+^ lymphocytes. (B) Scatter-gated lymphocytes from PBMC. (C) Scatter-gated monocytes from PBMC. (D) CD16^+^-purified blood neutrophils. Staining with control isotype matched antibodies is represented.
(4.23 MB TIF)

Figure S3 Surface expression of γδTCR/CD3 complex by human eosinophils following induction by a mycobacterial γδTCR ligand. (A–B) CD3, γδTCR, αβTCR and CD8 surface expression on EPO^+^-purified peripheral blood eosinophils incubated with culture medium (A) or with 40 nM TubAg for 2 h (B). Staining with control isotype matched antibodies is represented.
(3.02 MB TIF)

Figure S4 Surface expression of CD3, γδTCR and CD8 on cord blood-derived eosinophils. Eosinophils derived from CD34^+^ cord blood cells (day 21) were analysed for CD3, γδTCR and CD8 cell surface expression after gating on EPO^+^ cells. Staining with control isotype antibodies is represented.
(1.28 MB TIF)

Table S1 List, and characteristics of antibody used in this manuscript. Mb: Membrane staining. IC: Intracellular staining
(0.06 MB DOC)

Table S2 Gene-specific primer sequences
(0.04 MB DOC)
