# Supplementary figures and images for: A Functional γδTCR/CD3 Complex Distinct from γδT Cells Is Expressed by Human Eosinophils
Source: PLoS One. 2009 Jun 17;4(6):e5926. doi: 10.1371/journal.pone.0005926 (PMC2693924; doi:10.1371/journal.pone.0005926)

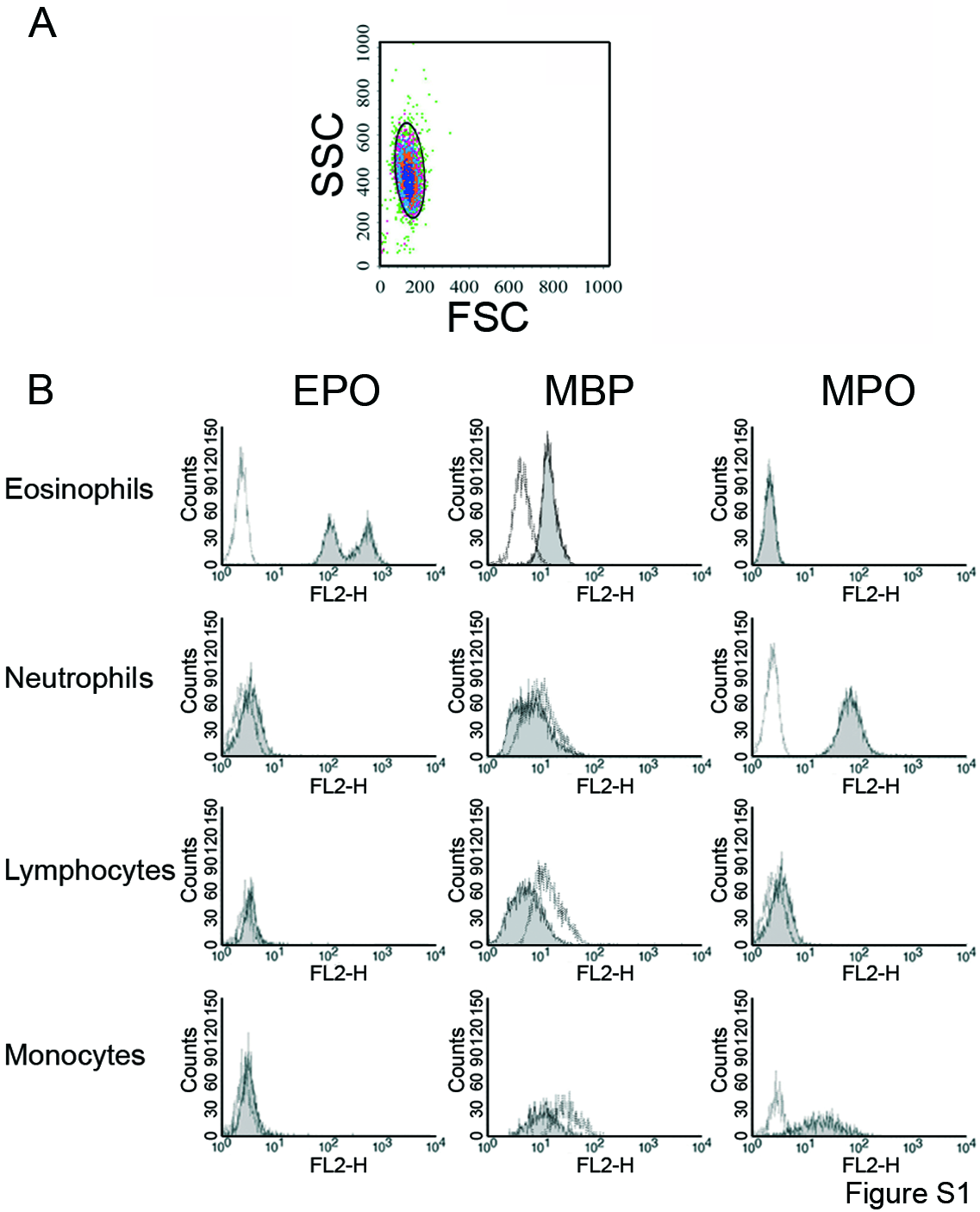

Supplement: Figure S1 — Gating strategy and specific identification of purified peripheral blood eosinophils in flow cytometry analysis. (A–B) Gating of purified peripheral blood eosinophils on forward and side scatter parameters immediately after purification. (A) Ellipsoids circle the analyzed population. (B). Specificity of eosinophil identification by intracellular detection of EPO, MBP or MPO using PE- or biotin-conjugated specific antibodies (grey shaded histograms). Staining on purified neutrophils as well as gated lymphocytes and monocytes from purified PBMC is shown as control. Staining with control isotype matched antibodies is represented in white histograms. (6.41 MB TIF) [file pone.0005926.s001.tif]

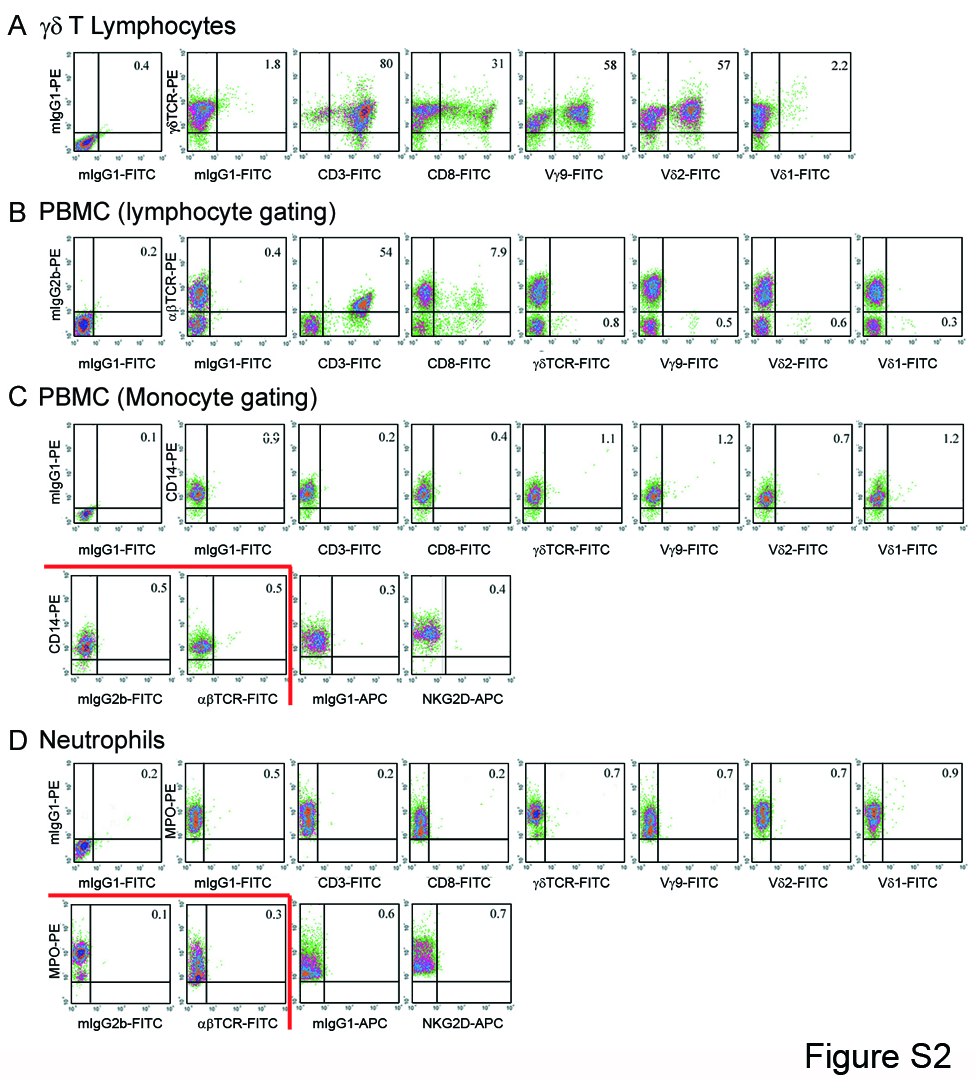

Supplement: Figure S2 — Surface expression of CD3, γδTCR, Vgamma9, Vdelta2,Vdelta1, αβTCR, CD8 and NKG2D detected by flow cytometry in double staining on immune cell populations. (A) In vitro-generated BrHPP-induced gamma9 delta2 TCR+ lymphocytes. (B) Scatter-gated lymphocytes from PBMC. (C) Scatter-gated monocytes from PBMC. (D) CD16+-purified blood neutrophils. Staining with control isotype matched antibodies is represented. (4.23 MB TIF) [file pone.0005926.s002.tif]

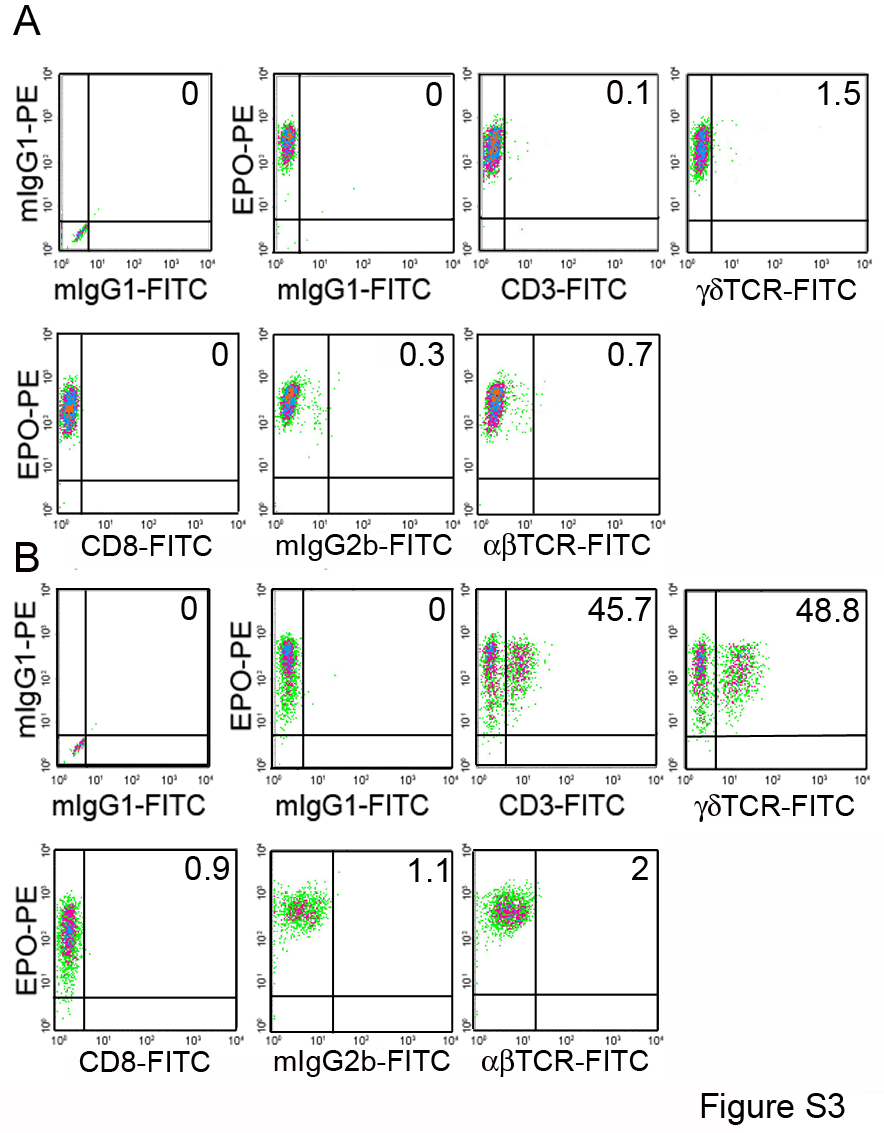

Supplement: Figure S3 — Surface expression of γδTCR/CD3 complex by human eosinophils following induction by a mycobacterial γδTCR ligand. (A–B) CD3, γδTCR, αβTCR and CD8 surface expression on EPO+-purified peripheral blood eosinophils incubated with culture medium (A) or with 40 nM TubAg for 2 h (B). Staining with control isotype matched antibodies is represented. (3.02 MB TIF) [file pone.0005926.s003.tif]

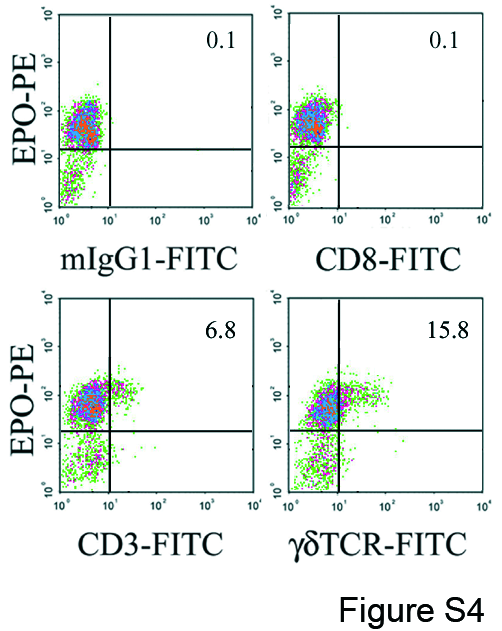

Supplement: Figure S4 — Surface expression of CD3, γδTCR and CD8 on cord blood-derived eosinophils. Eosinophils derived from CD34+ cord blood cells (day 21) were analysed for CD3, γδTCR and CD8 cell surface expression after gating on EPO+ cells. Staining with control isotype antibodies is represented. (1.28 MB TIF) [file pone.0005926.s004.tif]
