# Supplementary material for: A Functional γδTCR/CD3 Complex Distinct from γδT Cells Is Expressed by Human Eosinophils
Source: PLoS One. 2009 Jun 17;4(6):e5926. doi: 10.1371/journal.pone.0005926 (PMC2693924; doi:10.1371/journal.pone.0005926)
Supplement: Table S1 — List, and characteristics of antibody used in this manuscript. Mb: Membrane staining. IC: Intracellular staining (0.06 MB DOC) [file pone.0005926.s005.doc]

| ***Flow cytometry*** |  |  |  |  |
| --- | --- | --- | --- | --- |
| **Antibody** | **Clone** | **Supplier** | **Dilution** | **Isotype** |
| Anti-CD3-FITC (ε chain) | SK7 | Pharmingen | 1 :25 | mIgG_1_ |
| γδTCR-FITC (pan γδ TCR) (Mb) | B1 | Pharmingen | 1 :100 | mIgG_1_ |
| γδTCR-FITC (pan γδ TCR) (IC) | Immu510 | Immunotech | 1 :50 | mIgG_1_ |
| γδTCR-PE (pan γδ TCR) (Mb) | B1 | Pharmingen | 1 :10 | mIgG_1_ |
| Vγ9-FITC | B3 | Pharmingen | 1 :100 | mIgG_1_ |
| Vδ2-FITC | Immu389 | Immunotech | 1 :25 | mIgG_1_ |
| Vδ2-PE | B6 | Pharmingen | 1 :50 | mIgG_1_ |
| Vδ1-FITC | TS82 | Endogen | 1 :50 | mIgG_1_ |
| αβTCR-FITC (pan αβ TCR) | BMA031 | Serotec | 1 :25 | mIgG_2b_ |
| αβTCR-PE (pan αβ TCR) | BMA031 | Immunotech | 1 :25 | mIgG_2b_ |
| NKG2D-APC | 1D11 | Pharmingen | 1 :25 | mIgG_1_ |
| CD8-FITC | B911 | Pharmingen | 1 :50 | mIgG_1_ |
| CD14-PE | M5E2 | Pharmingen | 1 :50 | mIgG_2a_ |
| MBP | AHE-3 | Pharmingen | 1 :50 | mIgG_1_ |
| MPO-PE | 5B8 | Phrmingen | 1 :50 | mIgG_1_ |
| IgG_1_-FITC | MOPC-21 | Pharmingen |  |  |
| IgG_1_-APC | MOPC-21 | Pharmingen |  |  |
| IgG_1_-PE | DAK-G01 | Dako |  |  |
| IgG_1_-biotin | 4E3 | Beckman Coulter |  |  |
| IgG_1_ | MOPC-21 | Pharmingen |  |  |
| IgG_2b_-FITC | MCA691F | Serotec |  |  |
| IgG_2b_-PE | 27-35 | Pharmingen |  |  |
| IgG_2a_-PE | G155-178 | Pharmingen |  |  |
|  |  |  |  |  |
| ***Stimulation*** |  |  |  |  |
| **Antibody** | **Clone** | **Supplier** | **Concentration** | **Isotype** |
| Anti-CD3 (ε chain) | UCHT1 | Biolegend | 10µg/ml | mIgG_1_ |
| Anti-γδTCR (pan γδ TCR) | 5A6.E9 | Endogen | 10µg/ml | mIgG_1_ |
| Anti-Vδ1 | R9.12 | Immunotech | 10µg/ml | mIgG_1_ |
| Mouse IgG_1_ | B-Z1 | Diaclone | 10µg/ml |  |
| Anti-mouse IgG F(ab’)_2_ |  | Sigma | 10µg/ml |  |
| Secretory IgA |  | Sigma | 7.5µg/ml |  |
| Anti-IgA | NIF2 | Immunotech | 10µg/ml | IgA_1_ |
