# Supplementary material for: A Functional γδTCR/CD3 Complex Distinct from γδT Cells Is Expressed by Human Eosinophils
Source: PLoS One. 2009 Jun 17;4(6):e5926. doi: 10.1371/journal.pone.0005926 (PMC2693924; doi:10.1371/journal.pone.0005926)
Supplement: Table S2 — Gene-specific primer sequences (0.04 MB DOC) [file pone.0005926.s006.doc]

| **Primer** | **Sequence (5’-3’)** | **Annealing** |
| --- | --- | --- |
| Vδ1 | TCCAGCCTGCTGTGTGTATTTGTG | 60°C |
| Jδ3 | AAAAACATCTGTCGGGTGTCCCAG |
| Vδ1 | TCCAGCCTGCTGTGTGTATTTGTG | 60°C |
| Jδ4 | TCCTTTGCCAAACATCAGGGGTCT |
| Vδ2 | TCAACTGGTACAGGAAGACCCAAG | 57°C |
| Cδ | TGACAGCATTGTACTTCCCACTGG |
| VγI | GYTKTTCCCAYTGCAGCCAGTCAG | 58°C |
| JγP | CTTTGTTCCGGGACCAAATACC |
| CD8 | CTATACCTCTCCCAAAACAAgCCC | 56°C |
|  | CCCAGATGTAGATATCACAGGCGA |
| β_2_m (30 cycles) | CAGCGTACTCCAAAGATTCAGGT | 52°C |
|  | TGGAGACAGCACTCAAAGTAGAA |
| CD3γ | ATTTTCGTCCTTGCTGTTGG | 60°C |
|  | 5’GTTTCCTTGAAGGTGGCTGT |
| CD3δ | CGTTTCTCTCTGGCCTGGTA | 60°C |
|  | CCATGTGATGCTGGTATTGC |
| CD3ε | GGGGCAAGATGGTAATGAAG | 60°C |
|  | CCAGGATACTGAGGGCATGT |
| CD3ζ | GCTGGATGGAATCCTCTTCA | 60°C |
|  | GCCACGTCTCTTGTCCAAA |
| CD8α | ACTTGTGGGGTCCTTCTCCT | 60°C |
|  | GTCTCCCGATTTGACCACAG |
| TCRγ | 5’AACGGTGCCAGAAAAGTCAC | 60°C |
|  | TGTCTTTGGGATCCATTGTG |
| TCRδ | CTGTGCACTCCACTGACTTTG | 60°C |
|  | GGGTTTATGGCAGCTCTTTG |
| GAPDH | CGTCCCGTAGACAAAATGGT | 60°C |
|  | AGGTCAATGAAGGGGTCGTT |
